# CT Navigation-Assisted Transfacial Removal of Parotid Stones: Does It Work?

**DOI:** 10.3390/jcm14072338

**Published:** 2025-03-28

**Authors:** Michele Gaffuri, Ludovica Battilocchi, Matteo Lazzeroni, Lorenzo Pignataro, Pasquale Capaccio

**Affiliations:** 1 Department of Otolaryngology and Head and Neck Surgery, Fondazione IRCCS Ca’ Granda Ospedale Maggiore Policlinico, 20122 Milan, Italy; ludovica.battilocchi@policlinico.mi.it (L.B.); lorenzo.pignataro@policlinico.mi.it (L.P.); 2 Department of Clinical Sciences and Community Health, Dipartimento di Eccellenza 2023–2027, University of Milan, 20122 Milan, Italy; 3 Department of Otolaryngology, ASST Fatebenefratelli Sacco, 20157 Milan, Italy; matteo.lazzeroni@unimi.it (M.L.); pasquale.capaccio@unimi.it (P.C.); 4 Department of Biomedical, Surgical and Dental Sciences, University of Milan, 20122 Milan, Italy

**Keywords:** parotid stones, minimally invasive technique, CT navigation assisted, endoscopy assisted, sialolithiasis

## Abstract

**Background/Objectives:** The failure rate of minimally invasive surgical approaches to parotid stones is about 10%, primarily due to the presence of large, impacted, or unpalpable deep stones. When stones are palpable and exceed 7 mm in size, a combined transfacial and sialendoscopic approach offers a safe and effective surgical option, while unpalpable and impacted stones located in the parenchyma, not visible or accessible through sialendoscopy, can be treated with a CT-guided transfacial approach. **Methods:** Twenty-two patients (three females, mean age 53 years, range 32–73 years) underwent CT navigation-assisted transfacial removal of unpalpable and impacted parotid stones at the Department of Otolaryngology and Head and Neck Surgery of Fondazione IRCCS Ca’ Granda Ospedale Maggiore Policlinico of Milan between 2017 and 2024. **Results:** The mean size of the stones was 7.4 mm (range 4–14 mm), while the mean depth of stones, calculated as the distance from the skin surface, was 8.7 mm (range 4–14 mm). Stones were removed successfully in all but five patients (77% success rate). Failure of the procedure was significantly associated (*p* < 0.05) with the depth of the stone (>12 mm); in all these cases, patients were treated immediately by means of traditional parotidectomy. **Conclusions:** The CT-navigation-assisted transfacial approach can be considered a safe, reliable, and efficacious option for the treatment of difficult unpalpable parotid stones, impacted and deeply located in the gland parenchyma. Stones deeper than 10 mm can be more effectively treated by means of traditional parotidectomy if extracorporeal lithotripsy is not available.

## 1. Introduction

The management of parotid stones has undergone significant evolution over the past few decades, reflecting advancements in medical technology and a growing emphasis on patient-centered care. Traditionally, the treatment of parotid stones relied heavily on invasive surgical procedures, such as sialadenectomy, which involves the complete removal of the parotid gland. This approach was particularly common in cases involving main duct or intraparenchymal stones, where the stones were deeply embedded within the glandular tissue [1]. While effective in addressing the stones, these traditional methods were associated with considerable drawbacks, including the risk of facial nerve injury, visible scarring, and extended recovery periods [2]. These complications often led to significant patient discomfort and functional impairments, prompting the need for less invasive alternatives.

In response to these challenges, the past two decades have witnessed a paradigm shift toward conservative and minimally invasive techniques. These newer approaches aim to preserve the integrity and function of the parotid gland while minimizing patient morbidity. Advances in imaging technology, such as ultrasound and sialendoscopy, have played a pivotal role in this transition, enabling clinicians to accurately diagnose and localize stones without resorting to invasive procedures. Sialendoscopy, in particular, has emerged as a cornerstone of modern parotid stone management, allowing for the direct visualization and removal of stones through small, natural ductal openings [1]. This technique is especially beneficial for stones located in the distal or proximal ducts, where they can often be extracted with minimal tissue disruption.

The choice of treatment modality depends on several factors, including the size, location, number, and mobility of the stones. For instance, minimally invasive surgical approaches to parotid stones, such as intra- or extracorporeal shockwave lithotripsy and interventional sialendoscopy, may be useful for stones smaller than 7 mm, but have a failure rate of approximately 10%, primarily for large, impacted stones [3]. These larger stones often pose significant challenges due to their immobility and the difficulty of extracting them through the narrow salivary ducts. In such cases, purely endoscopic methods may prove insufficient, necessitating alternative strategies to achieve successful stone removal while minimizing patient morbidity.

To address these challenges, a hybrid approach combining external and endoscopic surgery has been proposed as a less invasive alternative to traditional parotidectomy [4]. This technique aims to preserve the parotid gland while effectively removing stones that are difficult to treat with purely endoscopic methods. By integrating the precision of endoscopic visualization with the accessibility of external surgical techniques, this hybrid approach offers a balanced solution that reduces the risks associated with more invasive procedures while maintaining high success rates.

The integration of external and endoscopic techniques is not a novel concept. Early innovators like Baurmash and Dechiara [5] pioneered extra-oral sialolithotomy without parotidectomy, using plain radiographs and high-definition ultrasound to locate and remove stones. This marked a significant shift away from traditional gland removal. Similarly, Nahlieli et al. [4] advanced the field by combining ultrasonography and sialendoscopy to identify stones, which were then extracted through a small vertical skin incision. This method proved that stone removal could be achieved with minimal tissue disruption.

Overtonet al. [2] further refined this hybrid approach by using a salivary endoscope to locate stones, followed by surgical extraction via a pre-auricular incision. This technique reduces tissue damage and lowers the risk of complications such as facial nerve injury and scarring, which are common in traditional parotidectomy. By preserving the parotid gland and its function, this hybrid method represents a major step forward in treating complex parotid stones.

This innovative strategy not only improves clinical outcomes, as evidenced by multiple studies [6,7,8], but also enhances patient satisfaction by reducing recovery times and preserving glandular function, reducing morbidity and avoiding the risks associated with more invasive parotidectomies, which carry well-documented complications [2].

This combined approach has also been described for removing parotid foreign bodies [9] and was adapted during the COVID-19 pandemic with appropriate precautions to minimize contamination risks [10]. However, not all parotid stones are palpable or accessible via sialendoscopy. In such cases, extracorporeal lithotripsy, supported by real-time ultrasonographic monitoring [11], was historically a safe and effective therapeutic option. Unfortunately, the availability of extracorporeal lithotripters has significantly declined due to commercial challenges, leaving the international medical community seeking alternatives to parotidectomy for managing deep, unpalpable stones inaccessible to sialendoscopy.

Recent advancements in imaging-guided techniques have provided new solutions for challenging cases of obstructive parotid disease. We recently described a technical note on treating such stones using a CT-guided transfacial approach, followed by combined CT- and MRI-guided sialodochoplasty to address Stensen’s duct stenosis and megaduct [12]. For unpalpable, impacted parenchymal stones—particularly those with deep Stensen’s duct kinking or strictures that are not visible or accessible via sialendoscopy—CT-guided assistance offers a viable treatment option [13,14]. Building on our preliminary technical description [13], we present our experience in managing these complex cases using a CT-navigation-assisted transfacial approach.

## 2. Materials and Methods

### 2.1. Patients and Indications

Between October 2017 and September 2024, 22 patients (3 females; mean age 53 years, range 32–73 years) underwent CT-navigation-assisted transfacial removal of symptomatic, unpalpable, and impacted parotid stones at the Department of Otolaryngology and Head and Neck Surgery, Fondazione IRCCS Ca’ Granda Ospedale Maggiore Policlinico, Milan. Initial clinical evaluation, including visual inspection and palpation of the parotid region, revealed no causes of salivary obstruction. All patients underwent ultrasound (US) assessment (Hitachi H21 system 7.5 MHz, Hitachi High Technology Corporation Ltd., Tokyo, Japan), which identified the presence of stones, followed by a CT scan (GE LightSpeed 64 Slice CT scanner, GE Medical Systems, Waukesha, WI, USA) to confirm the exact location and size of the stones.

Sialendoscopy was performed under local anesthesia using a 0.8 mm Nahlieli sialoendoscope (Karl Storz Co., GmbH, Tuttlingen, Germany) after widening the papilla of Stensen’s duct with salivary probes (Bowman probes, Karl Storz, Tuttlingen, Germany). In all cases, the stones were neither visible nor accessible via sialendoscopy.

Inclusion criteria consisted of the following:

- Unpalpable, impacted parotid stones deeply located within the gland parenchyma, rendering them invisible or unreachable by sialendoscopy.
- Recurrent symptoms such as pain, parotid swelling during meals, and recurrent sialadenitis or abscess.

Exclusion criteria included the following:

- Palpable parenchymal stones accessible via sialendoscopy and treatable with conventional sialendoscopy-assisted transfacial approaches.

Before surgery, all patients provided informed consent regarding the potential risks and complications of the procedure, including the possibility of conversion to traditional parotidectomy if the imaging-guided approach failed.

### 2.2. Surgical Technique

**Figure 1:**
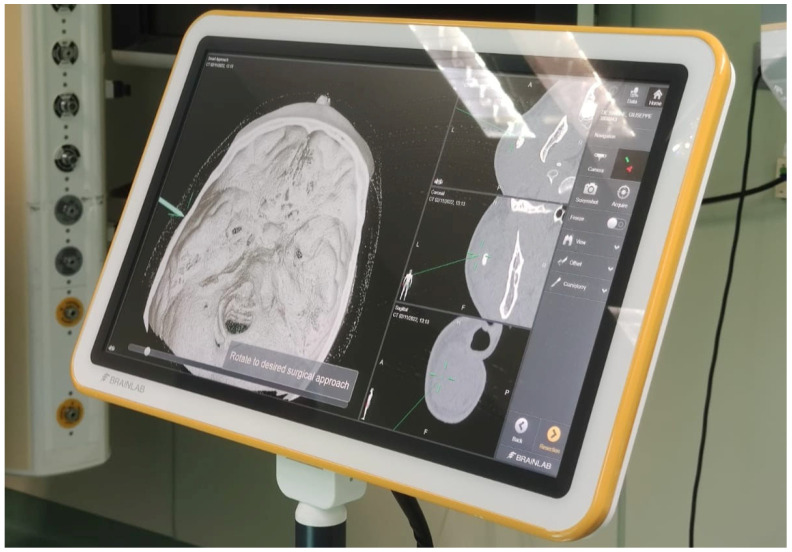
Before surgery, DICOMs were imported on iPlan CMF software (Brainlab, Munich, Germany) to set up the optical-based navigation system of the operating room.

**Figure 2:**
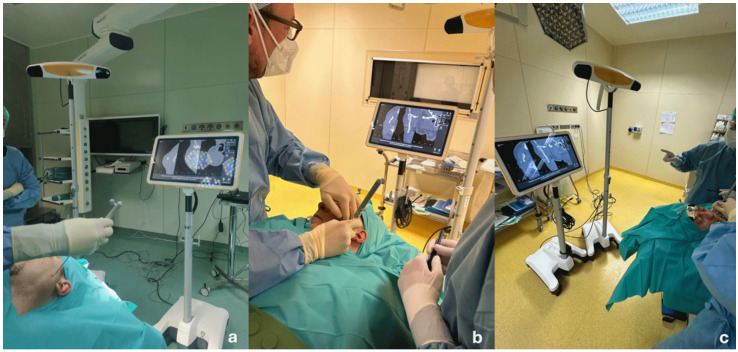
Optical-based navigation was used to locate the stone in the gland parenchyma (**a**–**c**).

**Figure 3:**
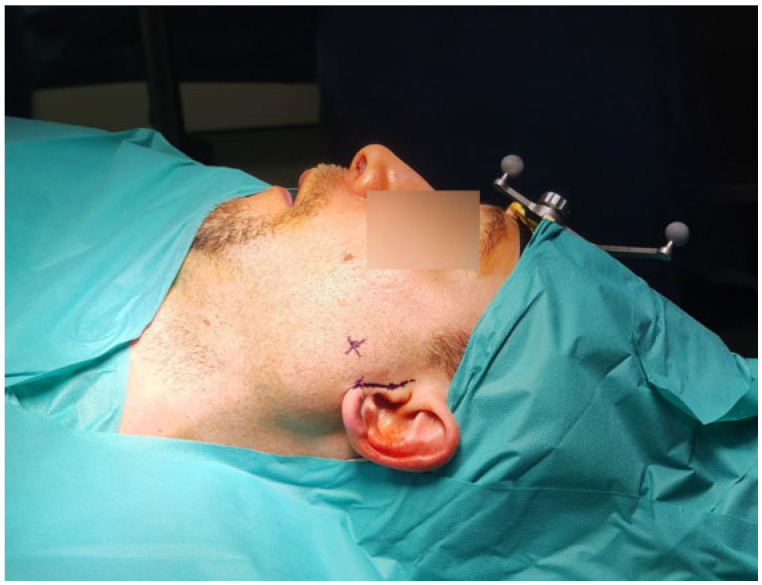
Stone position was marked on the skin surface.

**Figure 4:**
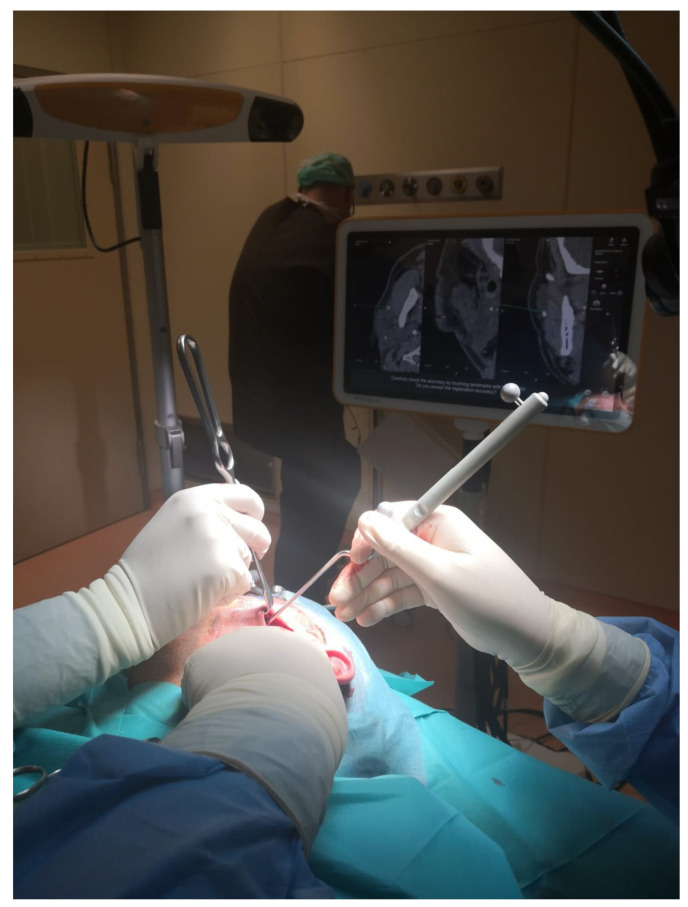
A blunt dissection, guided by the navigation system, allowed the exposure of the parotid gland and the subsequent exact localization of the stone as visualized on the display.

**Figure 5:**
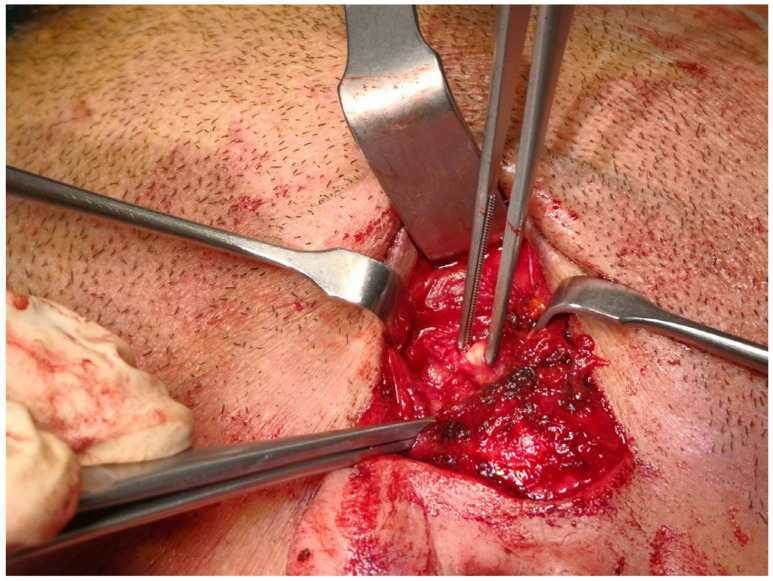
The duct was then incised and opened under navigation guidance and the stone removed.

Before surgery, the patients’ digital imaging and communications in medicine data-set (DICOM) were imported on iPlan CMF 3.0 software (Brainlab, Munich, Germany) to set up the optical-based navigation system of the operating room (Figure 1). A reference star array was attached to a headband on the head of the patients and a laser pointer was used to record landmark points on the face. During surgery, axial, coronal, and sagittal CT images, previously uploaded and reconstructed, were displayed on a kick display (Brainlab, Munich, Germany) to track the navigation pointer. The optical-based navigation was used to locate the stone (Figure 2) and its position marked on the skin surface (Figure 3). A preauricular skin incision was made, a skin flap was raised, and a blunt dissection, guided by the navigation system, allowed the exposure of the parotid gland and the subsequent exact localization of the stone as visualized on the display (Figure 4). A neurostimulator (Neuro-Pulse^®^, Bovie Medical Corporation, Clearwater, FL, USA) was used to check the functioning of the branches of the VII cranial nerve. The duct was then incised and opened under navigation guidance, and the stone was removed (Figure 5) with dedicated dissectors; a further sialendoscopic exploration of the duct was made to exclude the presence of any residual stones, and a saline or antibiotic irrigation was made. The glandular acini were closed together and a hemostatic patch (Tabotamp^®^ Ethicon Sarl, Neuchatel, Switzerland) was placed over the incision; the parotid fascia and the skin incision were sutured. All patients maintained fasting for 48 h and a compressive dressing for at least 72 h; a peri-operative antibiotic prophylaxis was given (Table 1).

### 2.3. Post-Operative Follow-Up

All patients were clinically re-examined one week, one month, three months, and one year after the procedure; US assessment was performed one month after the procedure to check the restored morphology and echogenicity of the parotid gland.

### 2.4. Subjective Evaluation

A quality-of-life (QOL) questionnaire named the Chronic Obstructive Sialadenitis Symptoms (COSS) questionnaire [15] was proposed to all patients before surgery and was also obtained one month after the surgical procedure only from the patients treated successfully.

This self-administered questionnaire, made of 20 questions, was used to examine symptoms of chronic sialadenitis and their impact on patients’ lives, obtaining a final score between 0 and 100. According to Aubin-Pouliot et al. [15], a COSS score < 10 defines mild disease or complete resolution of symptoms, a COSS score of 10–25 defines moderate disease or partial resolution of symptoms, and a COSS score > 25 defines severe disease or non-resolution.

## 3. Results

Twenty-two patients (three females; mean age 53 years, range 38–75) underwent CT-navigation-assisted transfacial removal of parotid stones. The mean stone size was 7.4 mm (range 4–14 mm), with stones located in the right parotid gland in 6 patients and the left gland in 16 patients. All stones were clinically unpalpable and located within the gland parenchyma in a secondary ductal branch, as confirmed by ultrasound (US) and CT imaging. The Mann–Whitney test revealed no statistically significant association between stone diameter and surgical failures (*p* > 0.05). The mean depth of the stones, measured from the skin surface, was 8.7 mm (range 4–14 mm).

Stones were successfully removed in 17 of 22 patients (77% success rate). Surgical failure was significantly associated (*p* < 0.05) with a stone depth greater than 12 mm. In all five unsuccessful cases, patients underwent immediate traditional parotidectomy. The overall mean surgical time was 150 min (range 55–354 min); the mean surgical time in cases of success (stone removal) was 94 min (range 55–178 min), while that in cases of conversion to parotidectomy was 267 min (range 207–354).

No major intraoperative complications, including temporary or permanent facial nerve palsy, were observed. Postoperatively, early sialocele occurred in three cases, initially managed with 48 h of fasting and compressive dressing. Two cases were resolved successfully with botulinum toxin (Xeomin, Merz Aesthetics, Raleigh, NC, USA) injection (30 IU) under US guidance 15 days post-surgery. The remaining case required traditional parotidectomy due to persistent sialocele. The overall parotidectomy rate was 27% (6/22).

Patients were discharged after a mean hospitalization of five days and evaluated clinically at one week and one month after surgery. US imaging performed one month after surgery in successfully treated patients confirmed restored salivary flow, evidenced by clinical flushing of saliva. The mean follow-up duration was 18 months (range 1–59 months). All patients were satisfied with their facial scars and remained symptom-free.

Preoperatively, 16 patients had a COSS score > 25, indicating severe disease, while 1 patient had a score between 10 and 25, indicating mild disease. The mean preoperative COSS score was 41.5 (range 18–82), which improved to a mean postoperative score of 7.0 (range 0–26).

## 4. Discussion

Unpalpable parotid stones smaller than 7 mm have traditionally been managed with extracorporeal shock wave lithotripsy (ESWL) under ultrasound guidance [1] or sialendoscopy-assisted intraductal shock wave lithotripsy (ISWL), using either pneumatic [16] or laser [1] systems. Currently, EWSL is no longer available due to limitations in factory productions, while laser ISWL remains available, but only one pneumatic lithotripter is accessible for sialendoscopy-assisted procedures [17]. Unpalpable and impacted stones, particularly those inaccessible via sialendoscopy due to duct kinking or strictures, represented a significant therapeutic challenge until the development of the CT-navigation-assisted transfacial approach in 2018 [13].

While the conventional transfacial approach for palpable stones larger than 7 mm is well-documented, with high success rates and minimal complications, locating unpalpable stones has historically been a limitation, increasing the risk of facial nerve injury during dissection. In our study, 22 patients were treated with this novel approach, representing the largest reported series in the literature. The mean overall surgical time was 150 min (range: 55–354 min) and in particular, in the case of surgical success, intended as immediate stone removal, the mean surgical time was 94 min (range 55–178 min), demonstrating that this imaging-assisted approach does not negatively impact surgical efficiency. Notably, operative times improved with experience, decreasing to a minimum operating time of 55 min. On the contrary, in the case of unsuccessful procedures, with consequent conversion to parotidectomy, the mean surgical time was 267 min (range 207–354), consisting of the attempt to retrieve the stone by means of the CT-guided approach plus the subsequent parotidectomy.

The overall success rate for stone removal was 77% (17/22 patients), consistent with previous findings. Two additional case series have been reported, detailing outcomes for two and five patients, respectively [14,18]. Anicin et al. [14] described a 50% success rate for two patients with stones measuring 5 mm and 7 mm in diameter, located at depths of 72 mm and 65 mm. Focque et al. [18] reported a 100% success rate for five patients, with a mean stone size of 11 mm (depth not specified).

In our series, the mean stone size was 8.7 mm (range: 4–14 mm), with a mean size of 8 mm in failed cases, suggesting that size may influence success, though this correlation was not statistically significant. Failures were associated with stones deeper than 12 mm, necessitating conversion to traditional parotidectomy. This relationship between stone depth and failure was statistically significant (*p* < 0.05); for this reason, patients gave consent to both procedures, as the CT navigation system’s precision diminishes beyond 10 mm from the skin surface. In such cases, surgical dissection can alter gland anatomy, modifying CT coordinates and potentially displacing stones, complicating retrieval, particularly during reoperations. Anicin et al. [14] observed that without intraoperative CT updates, real-time corrections are impossible, making CT navigation ideal for fixed stones but less reliable for potentially mobile ones. In such cases, experienced surgeons may opt for ultrasound guidance, though this technique is less standardized and more operator-dependent [11]. The use of CT navigation in the management of parotid stones, particularly in cases involving unpalpable and deeply located stones, presents both advantages and challenges that must be carefully considered. One of the primary concerns is the amount of radiation exposure associated with obtaining radiological images for diagnostic and surgical planning purposes [19]. In the present case series, the use of CT was partially justified by the complexity of the cases, as all patients had unpalpable and deep parotid stones. In such scenarios, traditional ultrasound may not provide sufficient detail, especially in cases of recurrent infections or abscesses, where precise anatomical localization is critical for effective treatment. While magnetic resonance imaging (MRI) is a radiation-free alternative, it is not always feasible due to factors such as cost, availability, or patient contraindications.

To mitigate the risks associated with radiation exposure, cone beam CT (CBCT) has emerged as a promising alternative to traditional maxillofacial CT. CBCT delivers a significantly lower dose of radiation while still providing high-resolution images, making it a safer option for patients [20]. However, the integration of CBCT with existing neuronavigation systems, whether optical or magnetic, remains a challenge. For CT navigation to be widely adopted, it is essential that CBCT imaging is compatible with the neuronavigation systems currently available on the market. This would ensure that the benefits of reduced radiation exposure can be fully realized without compromising surgical precision.

Another critical consideration is the cost associated with CT navigation technology. Compared to traditional ultrasound-assisted procedures, the expenses related to CT navigation are undeniably higher. However, these costs can be more justifiable when viewed in the broader context of its applications across various head and neck subspecialties [21]. For instance, neuronavigation systems are not only used for the management of salivary stones but also play a crucial role in endoscopic sinus surgery and skull base surgery [22], both anterior and lateral. By sharing the cost of this technology across different disciplines, the financial burden can be distributed, making it a more viable investment for healthcare institutions.

While CT navigation offers significant advantages in the diagnosis and treatment of complex parotid stones, its use must be balanced against concerns related to radiation exposure, compatibility with existing systems, and cost. The adoption of CBCT as a lower-radiation alternative and the shared utilization of neuronavigation technology across multiple surgical specialties can help address these challenges. As the field continues to evolve, further advancements in imaging and navigation systems will likely enhance the feasibility and accessibility of these techniques, ultimately improving patient outcomes in the management of parotid stones and other head and neck conditions.

No major complications were observed in our case series. Intraoperative nerve monitoring was essential to minimize the risk of facial nerve injury, particularly for stones in the middle and distal thirds of Stensen’s duct, where the nerve is less protected. Three patients (13.6%) developed parotid sialoceles, a slightly higher rate than the 10% reported in traditional approaches [23], likely due to prior parotid abscesses and duct stenosis. Two cases had been cured with botulinum toxin injections and compressive dressing, while one required total parotidectomy after conservative measures failed. Botulinum toxin therapy still represents a viable option for managing sialoceles or salivary fistulas, alongside conservative and surgical interventions. The total conversion rate to traditional parotidectomy was 27% (6/22). Although this may appear high, it underscores that this minimally invasive approach is currently the only option for treating discrete, unpalpable intraparenchymal stones. Preoperatively, 21 of 22 patients had severe disease (COSS score > 25), reflecting the significant impact on quality of life and the need for effective, minimally invasive treatments. One month post-surgery, the COSS questionnaire, administered to patients with successful outcomes, indicated symptom resolution (COSS score < 10) in 16 patients (*p* < 0.0041), with the most notable improvement in swelling frequency between meals. Only one patient, who later underwent parotidectomy due to inadequate response to botulinum toxin, experienced worsened symptoms (COSS score > 25).

## 5. Conclusions

The CT-navigation-assisted transfacial approach is a safe, reliable, and efficacious option for treating difficult, unpalpable parotid stones that are deeply impacted within the gland parenchyma. Although CT navigation requires greater technical effort, includes additional costs, and involves patient radiation exposure, this novel technique often avoids the need for traditional parotidectomy, along with its associated complications. For unpalpable parenchymal parotid stones located deeper than 10 mm, traditional parotidectomy remains a more effective treatment option when extracorporeal lithotripsy is unavailable. Larger patient series are needed to validate these preliminary findings.

## Figures and Tables

**Table 1:** Patient demographics, clinical findings, details of parotid stones, and outcomes.

Patient Demographics, Clinical Findings, Details of Parotid Stones and Outcomes	
Total Patients (n)	22
Gender (M:F)	19:3
Age (years; mean [range])	53 [38–75]
Total Glands (n)	22
Side affected (L:R)	16:6
Stone size (mm; mean [range])	7.4 [4–14]
Stone depth (mm: mean [range])	8.7 [4–14]
Surgical time (minutes; mean [range])	150 [55–354]
Stones successfully removed (n/n [percentage])	17/22 [77%]
Failures (n/n [percentage])	5/22 [23%]
Depth of stones in failures (mm; mean [range])	12 [11–14]
Postoperative sialocele (n/n [percentage])	3/22 [14%]
Total parotidectomy rate (n/n [percentage]	6/22 [27%]
Follow-up (months; mean [range])	18 [1–59]
COSS preoperative (median [range])	41.5 [18–82]
COSS postoperative (median [range])	7 [0–26]

COSS = chronic obstructive sialadenitis symptoms.

## Data Availability

The original contributions presented in this study are included in the article. Further inquiries can be directed to the corresponding author.
